# The Rate of Concentration Change and How It Determines the Resolving Power of Olfactory Receptor Neurons

**DOI:** 10.3389/fphys.2016.00645

**Published:** 2016-12-27

**Authors:** Harald Tichy, Maria Hellwig, Lydia M. Zopf

**Affiliations:** Department of Neurobiology, Faculty of Life Sciences, University of ViennaVienna, Austria

**Keywords:** electrophysiological recordings, ON and OFF olfactory receptor neurons, ramp-like concentration changes, resolving power, speed acurracy trade-off

## Abstract

The response characteristics of olfactory receptor neurons (ORNs) and their corollary, the differential sensitivity and the resolving power, are fundamental to understand olfactory coding and the information extracted from a fluctuating olfactory signal. Previous work has focused on the temporal resolution of odor pulses presented for very brief periods at varying concentrations. The time course of the odor pulses as a stimulus parameter has not been considered. The present study investigated the precision of the ON and OFF ORNs on the antennae of the cockroach to discriminate increments and decrements of continuously rising and falling odor concentrations. Stimulation consisted of ramp-like upward and downward concentration changes in a trapezoid fashion. By varying ramp steepness, we examined the effect of the rate of concentration change. Both ORNs were clearly dependent on continuously rising and falling odor concentrations. As the rate of upward and downward concentration changes increases, differential sensitivity improves. Since the scatter of responses around the stimulus-response functions also increases, the resolving power for concentration increments and decrements deteriorates. Thus, the slower the rate of concentration change, the higher the precision in differentiating small concentration changes. Intuitively, the inverse relationship between the rate of concentration change and the resolving power is not surprising because accuracy requires time. A high degree of precision at slow concentration rates enables the cockroach to use information about the onset and offset slopes of odor pulses in addition to the pulse height to encode the spatial-temporal structure of turbulent odor plumes.

## Introduction

Olfaction is generally thought to be a “slow” sense compared to “fast” senses such as vision and hearing (Laurent, 1999). Decades of research—using behavioral analysis and electrophysiological recordings—have led to the broad consensus that many insects and marine crustaceans tracking a turbulent odor plume are able to detect and respond to the intermittent pattern of the odor signal. Although there are several variations within this framework, almost all studies have focused on the speed or the rate of arrival of the odor signal as the key variable of the data. Nothing is known about the accuracy with which the concentration of the intermittent odor signal is determined. We address this question in the present study.

Odors from food or mates are carried by wind or water in plumes characterized by turbulence of the medium. Turbulence causes the plume to break up into discrete patches or pulses of odor of varying concentration interspersed with clean air or water (Murlis and Jones, 1981; Zimmer-Faust et al., 1988, 1995; Moore and Atema, 1991). The intermittent pulses in a turbulent odor plume constitute a potentially powerful navigational cue to an organism searching for an odor source. This is especially true when combined with assistive cues such as flow direction and speed (reviewed in Webster and Weissburg, 2001; Moore and Crimaldi, 2004; Koehl, 2006; Riffell et al., 2008). The concentration of an odor pulse attenuates with increasing distance from the source. Moreover, the time periods of zero odor concentration (off-time) become longer with increasing distance from the source. These two features of the odor signal play fundamental roles in determining the overall temporal and spatial distribution of olfactory information (Murlis et al., 1992).

The initial step in olfaction is the instantaneous intercept of the odorant molecules in air or water dilution with the olfactory receptor neurons (ORNs). In almost all physiological experiments, this step was imitated by pulsing rapidly odor-laden air onto the antenna. Some electrophysiological studies have directly examined the maximum frequency of pulse resolution by ORNs. The pulse rates that reveal distinct bursts of action potentials are species specific and range from 5 to 50 Hz (Weissburg, 2000; Szyszka et al., 2014). In the cockroach, ORNs could follow 25-ms pulses of 1-hexanol up to rates of 40 Hz and 50-ms pulses of coconut oil up to 20 Hz (Lemon and Getz, 1997). Electroantennogram (EAG) recordings, which represent the summed potential of all electrical activity of an intact but excised antenna, revealed a maximum resolution of 3-ms pulses of 2-heptanone at 50 Hz in the hissing (orange spotted) cockroach and 125 Hz in the honey bee and locust (Szyszka et al., 2014).

In a series of brief odor pulses with equal concentration did only rarely each pulse evoke identical responses. At high repetition rates, the bursts of action potentials obtained in single unit recordings decreased in frequency (Lemon and Getz, 1997), and the amplitudes of summed responses in EAG recordings decreased (Szyszka et al., 2014). This decline in response may be due to adaptation or fatigue, suggesting that a high temporal resolution of odor pulses is not necessarily linked with highly accurate pulse concentration detection. The ORNs might be optimized to respond to transient concentration changes rather than to encode odor concentration. Acquiring precise information about odor concentration, as it relates to the spatial-temporal distribution in an odor plume, could be restricted to somewhat slower repetition rates. Since it is unlikely that ORNs have evolved to encode the frequency of pulsatile odor stimuli, a specific parameter related to the pulse rate may affect the response magnitude.

Measuring the instantaneous distribution of chemical signals in aquatic environments shows that, in addition to the intermittent plume structure, the peak heights and onset slopes of the odor pulses vary systematically along both the transverse and longitudinal axis of odor plumes (Atema, 1996; Moore and Crimaldi, 2004). With increasing distance from the source, odor plumes tended to spread horizontally, and vertically, and both mean peak height and slope of odor fluctuations decreased. The pulse onset slope and the correlated pulse height provide the strongest spatial gradients in turbulent odor plumes (Moore and Atema, 1988, 1991; Zimmer-Faust et al., 1995; Finelli et al., 1999). Lobsters can detect and use the shapes of odor pulses to determine their distance and position with regard to an odor source (Atema, 1985, 1996; Moore and Atema, 1991). ORNs specialized in detecting the pulse onset slopes and repetition rates of turbulent odor plumes passing over the lobster's chemoreceptor organs were considered to be best suited for mediating distance information (Gomez and Atema, 1996; Zettler and Atema, 1999).

Sharp odor pulses were not the optimal stimuli for studying the dependence of ORN responses on the pulse onset slope or the rate of concentration increase. It would be difficult if not impossible to control the rise time of transient concentration changes. Slow and continuous changes in odor concentration, in contrast, enable controlling the flow rate of the stimulating air stream and quantifying the rate of concentration change. Using this form of stimulation, we identified antagonistically responding ON and OFF ORNs on the cockroach's antenna. They are highly sensitive to two components of the odor stimulus: the instantaneous odor concentration and its rate of change (Hinterwirth et al., 2004; Tichy et al., 2005; Burgstaller and Tichy, 2012; Hellwig and Tichy, 2016). In both ORNs, gain control acts as a trade-off between sensitivity to instantaneous odor concentration and the rate of concentration change. When the concentration oscillates rapidly with brief periods, then adaptation improves the gain for instantaneous odor concentration and reduces gain for its rate of change. Conversely, when odor concentration oscillates slowly with long periods, adaptation increases gain for the rate of change at the expense of the instantaneous concentration.

The question tackled in the present study is mainly one of resolving power. What can be expected of the discharge when the ON and OFF ORNs are confronted with slow and continuous concentration changes? Once individual discharges become sufficiently different, the CNS can interpret them as showing a difference in odor concentration. How different must a pair of instantaneous odor concentrations be if the larger of the two responses is to accompany the larger of the two concentrations with a given probability? The issue of resolving power has been dealt with so far only in the cockroach's ON and OFF ORNs by testing upward and downward step-like changes in odor concentration, respectively (Burgstaller and Tichy, 2011). No data of this type are available for slow rates of concentration change or concentration gradients. Here we describe the effect of linearly ramped and damped concentration changes. By varying the onset and offset slopes, we tested a variety of rising and falling rates of concentration change. We determined the effect of the rate of concentration change on the accuracy of discriminating increments and decrements in odor concentration. Consistent with the general notion that olfaction is a slow sense, we found a trade-off between discrimination accuracy and the rate of concentration change in the ON and OFF ORNs: the slower the rate of odor concentration change, the finer the concentration discrimination.

## Materials and methods

### Preparation and recording

Adult male cockroaches (*Periplaneta americana*) were anesthetized with CO_2_ and placed on their dorsal surface in a closely fitted holder. Their body was immobilized by strapping it to the holder with Parafilm. For unobstracted stimulation with an air stream of changing odor concentration, the antenna was attached with adhesive tape and dental cement (Harvard Cement, Harvard Dental Gesellschaft Berlin) on the edge of a narrow ledge that extended from the holder. Then the cockroach was positioned in the experimental set-up, so that the odor delivery nozzle was about 10 mm away from the recording site on the antenna.

Recordings were made extracellularly between two electrolytically sharpened tungsten needles. The reference electrode was inserted lengthwise into the tip of the antenna, and the recording electrode at the base of the sensillum. The action potentials were amplified and band-pass filtered (0.1–3 kHz), passed through a 1401 plus A–D converter (Cambridge Electronic Design, UK), displayed on-line with the voltage output of the electronic flow meters on a monitor, stored on a hard disk and analyzed off-line using Spike2 software (Cambridge Electronic Design, UK).

### Occurrence and sensillum structure

The whip-like antenna of the cockroach *Periplaneta americana* consists of 120 to 180 ring-shaped segments which are covered on all sides by slanting bristles. They protect the multitude of olfactory sensilla beneath them from contact with any surface the antennae may encounter. The ON and the OFF ORNs occur together in the single-walled type *C* sensilla (*swC*; Schaller, 1978; Altner et al., 1983; Hinterwirth et al., 2004; Tichy et al., 2005). These sensilla resemble short, slightly curved hairs that taper to a sharp tip. About fifteen type *swC* sensilla are located on the distal and proximal margins of each antennal segment and make up 6% of the olfactory sensilla in the male cockroach. However, the single-walled type *C* sensilla are not the only sensilla that contain receptor neurons responsive to citrus fruit odor. Two physiological classes of single-walled type *B* sensilla, which comprise about 27% of the sensillum population, produce strong excitatory responses to lemon odor, in addition to a group of different natural food odors (banana, apple, orange, bread, meat and cheese). Thus, about four times the numbers of the single-walled type *C* sensilla (6%), which house the antagonistic ON and OFF ORNs, are associated with receptor neurons that are involved in lemon odor.

### Odor stimulus

Fruit odors are known to be highly effective stimuli for antennal olfactory receptor cells and antennal lobe neurons (Boeckh, 1974; Sass, 1978; Selzer, 1981, 1984). They contain a number of odor compounds belonging to different chemical classes (Günther, 1968; Shaw, 1979). The quality of odor compounds in natural fruits can differ considerably depending upon the region of origin, maturity and storage. We therefore used synthetic lemon oil (Roth, D ~ 0.85, Art. 5213.1) as a standardized fruit odor stimulus.

### Dilution flow olfactometer

Odor stimulation was provided by using an air dilution flow olfactometer in which odor concentration is varied by changing the ratios of the volume flow rates of clean and odor saturated air (Burgstaller and Tichy, 2011). Compressed air entering the olfactometer was cleaned and divided into two streams. Their flow rates were equalized by matching their rates in Rotameter type flow meters. The first air stream was bubbled out through hundreds of small holes in polyethylene tubing anchored at the bottom of a 25-l tank containing 100 ml of liquid odor of lemon oil. The second air stream was led through an empty control tank of the same design and remained clean. After emerging from the tank, each air stream was passed through an electrical proportional valve (Kolvenbach KG, KWS 3/4) and an air flow sensor (AWM; Honeywell). The two streams were then united. Ramp-like concentration changes were produced by opening linearly one valve and closing the other valve at the same rate. Thus, the total flow rate of the combined air stream was held constant at 1.5 m/s as the underlying odor/clean-air ratio varied. For stimulation, the mixed air stream was directed toward the antenna by way of a glass tube 7 mm in diameter. The air around the antenna was continually removed by a suction tube adjusted to a suction speed of 2 m/s. Stimulus concentration was calculated using the flow rate ratio of odor-saturated air to clean air and indicated throughout by the percent of the saturated air in the stimulus air stream leading to the antenna. “0% saturated air” means clean air only: the air stream directed onto the antenna contains no odor stimulus. “100% saturated air” refers to pure odorized air (stimulus air not mixed with clean air).

### Response evaluation

Impulse frequency (imp/s) was determined by impulse counts for fixed periods of time. These periods (bin widths) varied depending on the rate of concentration change. Impulse frequencies for ramps of 5%/s were counted during 0.5 s intervals, yielding 40 measurements for a 20 s ramp. The bin width for ramps of 20%/s was 0.2 s, resulting in 25 data points for a 5 s ramp, and the bin width for 50%/s ramps was 0.1 s, providing 20 data points for a 2 s ramp. At a rate of 5%/s, odor concentration would change by only 2.5%/s during a single 0.5 s interval used for counting impulses. Since two concentration readings are needed to determine the amount of change an error approaching this difference could easily be made. The accuracy of the difference could be obviously enhanced by lengthening bin width, but at the price of lengthening the time during which all changes other than that implied in the most general tendency would be ignored. This difficulty was gotten around by using running averages of groups of three. Starting with the first period, the mean for the first three periods was formed, then starting with the second period, the average for the next three was formed, then starting with the third, and so on. Each mean rate was taken as belonging to the midpoint of the middle period for which it was determined and was used to approximate the instantaneous value there (Burgstaller and Tichy, 2012).

The most important characteristics of a receptor neuron are probably their differential sensitivity and resolving power. We define differential sensitivity as the ratio of input to output or the mean change in frequency per unit change in stimulus magnitude. This quantity is given by the slope of the curve that approximates the relation between stimulus intensity and response. The resolving power of a receptor neuron can be determined from the differential sensitivity and the scatter of individual responses. We focused our attention on the minimum amount by which two odor concentrations must differ in order that a single receptor neuron with average differential sensitivity, in responding once to each of the two concentrations, will have a specified high probability (e.g., 90%) to give a larger response to the larger concentration. The question here is not a mean value as in differential sensitivity but rather what can be expected from a single pair of responses. A full mathematical development of the concepts underlying the resolving power (Δ*x*) was presented by Loftus and Corbière-Tichané (1981). The equation is

$$\begin{array}{l} \Delta x=\frac{\sqrt{2}\sigma }{\left|b\right|}\Phi^{-1}\left(y\right) \end{array}$$

in which |*b*| is the mean absolute slope of the stimulus-response functions. Because the slope of a parabola varies continuously along the curve and the parabolas approximating these functions were not the same for all ORNs, |*b*| was obtained by taking the mean of the individual slopes (i.e., first differential) corresponding to the stimulus actually presented. σ^2^ is the variance of the individual deviations of points about their respective regressions, *y* is the required probability (90%), and $\Phi_{\left(y\right)}^{-1}$ is the inverse of the distribution function of a standardized, normally distributed, random variable. $\Phi_{\left(0.9\right)}^{-1}$ = 1.28 (Diem and Lentner, 1970, Tables p. 28). In case of a linear regression, σ^2^ is estimated by

$$\begin{array}{l} \sigma^{2}=\frac{\sum \varepsilon^{2}}{n-2I},\text{and for a parabola by }\sigma^{2}=\frac{\sum \varepsilon^{2}}{n-3I}, \end{array}$$

where ε is the deviation of each individual point from its curve, *I* is the number of curves, and *n* the number of measurements. *n* is reduced by the number of degrees of freedom, which is *2I* because 2 estimates are necessary to determine each straight line (*a* and *b; y* = *a* + *bx*). Since the resolving power is calculated from parabolas, *n* is reduced by *3I*, corresponding to the 3 estimators for each parabola (*a, b* and *c; y* = *a* + *bx* + *cx*^2^).

This method can be applied if the following conditions are met: (*i*) the deviations of the individual points from their curves must be normally distributed about a mean of zero, and (*ii*) the absolute deviations (sign ignored) must not depend on the slope of the curves. The absolute deviations of single points from their regressions did not depend on the regression slopes. However, their distribution was not normal (*x*^2^-test). Though bell-shaped, the flanks of the distribution curve were too steep; the points tended to be located too centrally. This type of distribution will, if anything, underestimate the resolving power. The normal distribution model was accepted for the lack of a better one.

## Results

**Figure 1:**
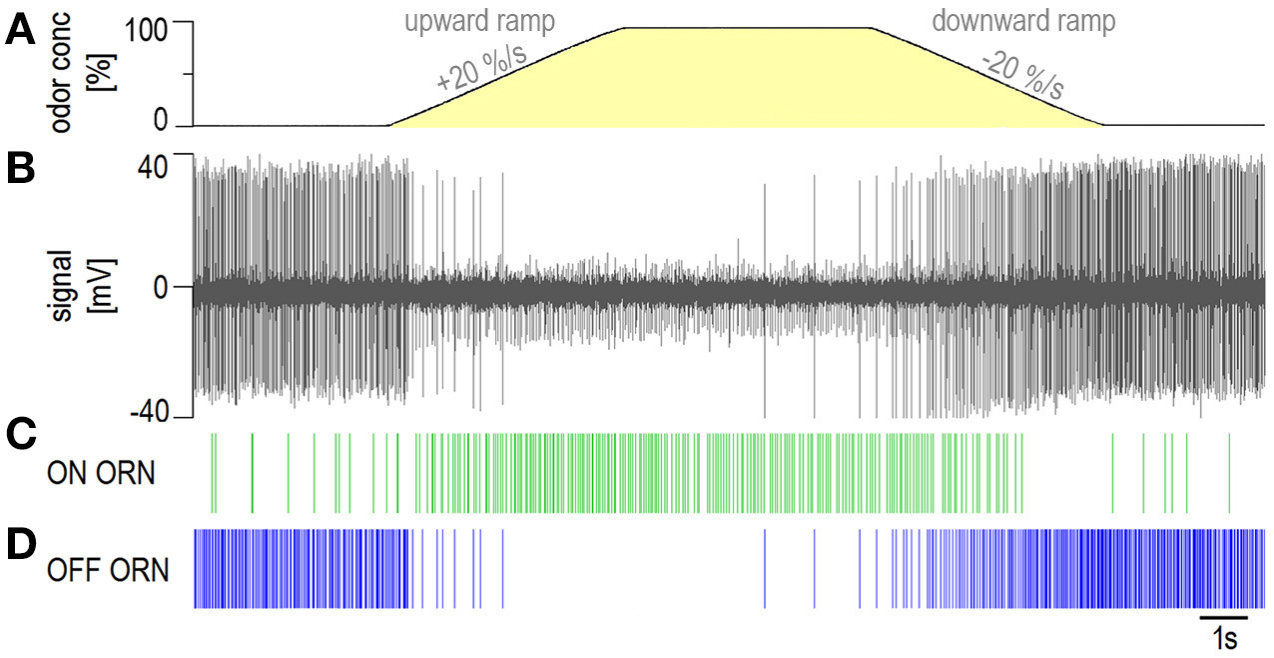
**Example of the activity of an ON and an OFF ORN in the same sensillum during a ramp-like upward concentration change of +20%/s followed by a ramp-like downward concentration change of −20%/s of the odor of lemon oil**. **(A)** Yellow region shows time course of odor concentration measured by flow meter. **(B)** extracellularly recorded activity; the OFF ORN displayed larger impulse amplitudes than the ON ORN. **(C,D)** responses of both types of ORNs represented in raster plots. Spike identification was performed offline using the software Spike 2.

The ON and OFF ORNs are combined in the same hair-like sensillum on the distal and proximal margins of each antennal segment. With rare exceptions, the recordings revealed the activity of both types of ORNs, clearly distinguishable by the amplitude and form of their impulses. The impulse amplitudes of the OFF ORN typically were larger than those of the ON ORN, and when the olfactory stimulus was absent, as expected, the OFF ORN fired at higher rates than the ON ORN (Hinterwirth et al., 2004; Tichy et al., 2005; Burgstaller and Tichy, 2011, 2012). The final identification of the two ORNs was based upon their opposite responses to upward and downward ramps in odor concentration. As shown by the single-sensillum recording in Figure 1, increasing the concentration at a constant rate of +20%/s raised impulse frequency in the ON ORN and lowered it in the OFF ORN. Correspondingly contrary effects can be produced by decreasing the odor concentration at a constant rate of −20%/s. The discharge rose progressively in both ORNs and lasted longer than the upward or downward concentration ramps. Furthermore, the ON-ORN's discharge was higher at the concentration plateau than at the interstimulus interval and conversely, the OFF-ORN's discharge was higher at the interstimulus interval than at the plateau phase.

**Figure 2:**
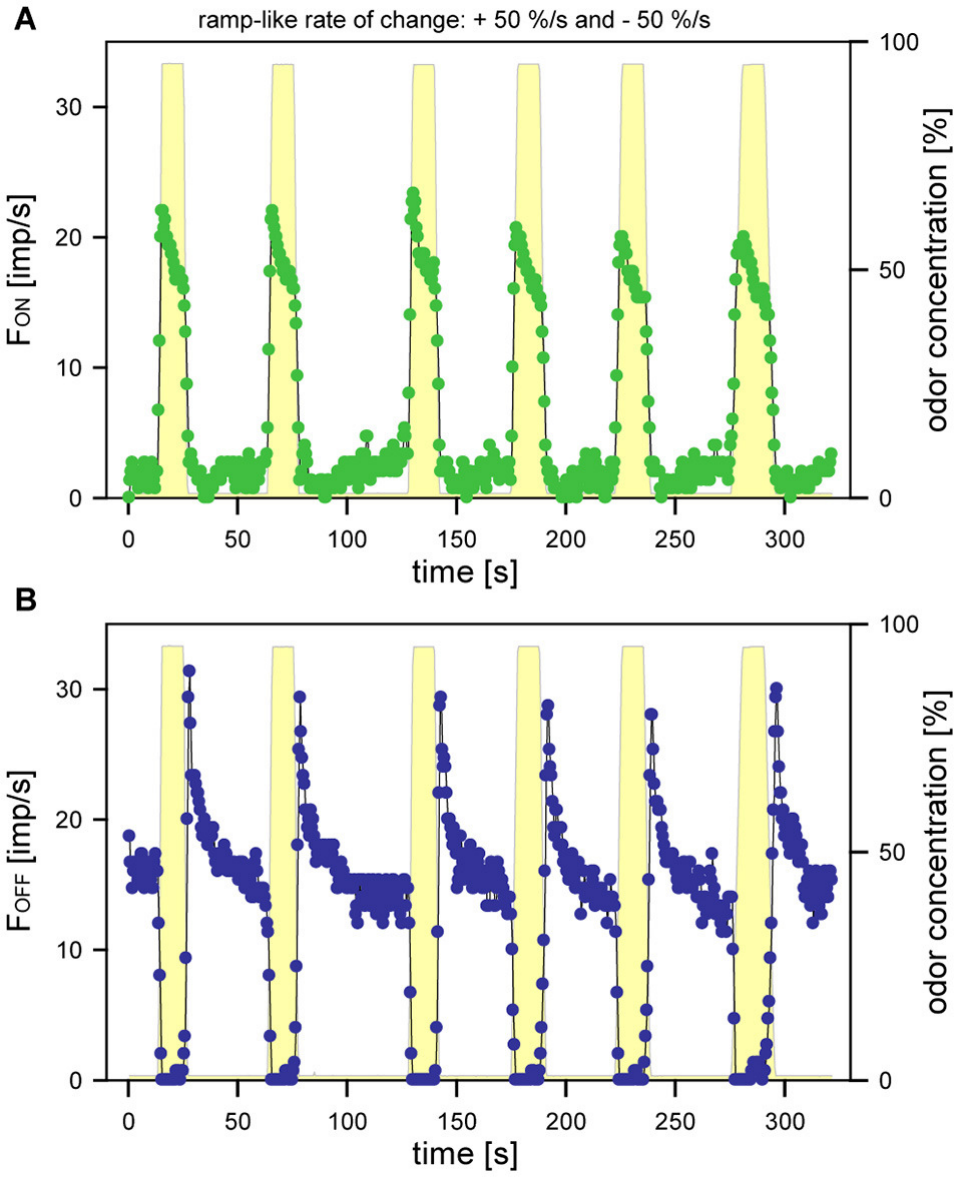
**Response profiles of an ON and an OFF ORN from the same sensillum during a series of 6 alternating upward and downward concentration ramps at a rate of +50%/s and −50%/s, respectively**. **(A)** responses of the ON ORN (*green*). **(B)** responses of the OFF ORN (*blue*). Yellow regions show time course of concentration change. Impulse frequencies determined for impulse counts of 0.1 s intervals.

As a necessary preliminary to experiments of the effect of the rate concentration change, the reproducibility of the responses was examined on 6 ON and 6 OFF ORNs. A typical example of a pair of ON and OFF ORNs to a series of upward concentration ramps of +50%/s alternating with downward concentration ramps of −50%/s is shown in Figure 2. The response profiles of each ORN changed very little during a series of experiments, but the response peaks displayed some variability. However, repeated observations of a single ORN had much less variability than single observations of many ORNs. Since repeated tests of single series of a given ramp would severely limit the number of different ramps to which an ORN could be subjected, two or three repetitions for each ramp an each ORN were accepted as a compromise.

**Figure 3:**
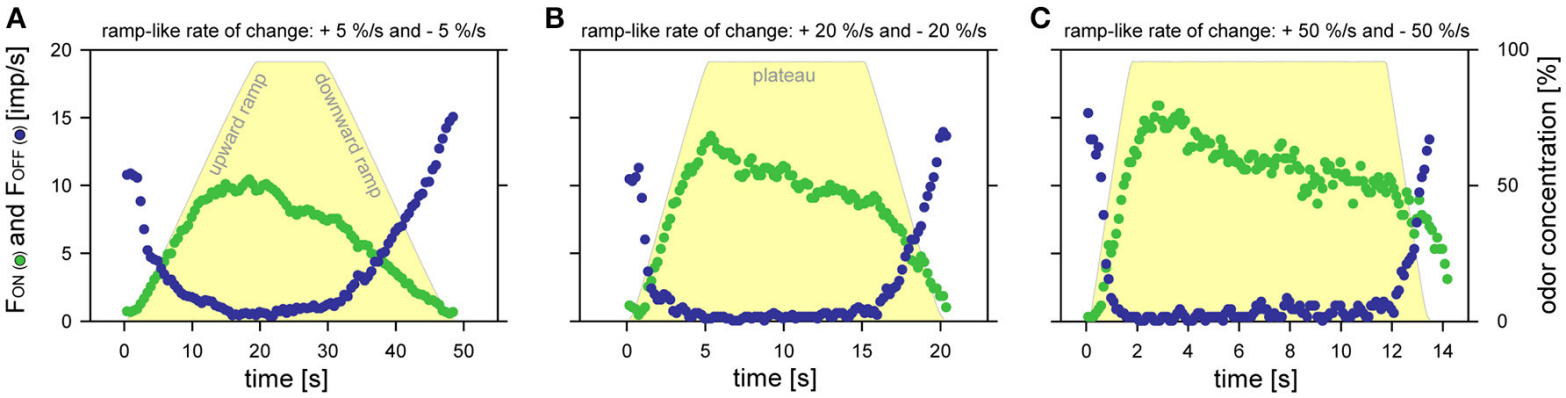
**Time course of odor concentration (***yellow regions***) and impulse frequency of an ON ORN (***green***) and an OFF ORN (***blue***) from the same sensillum**. Ramp-like upward and downward concentration changes at slow rates of 5%/s **(A)**, medium rates of 20%/s **(B)** and fast rates of 50%/s **(C)**. Bin widths for impulse counts were 0.5 s for 5%/s ramps, 0.2 s for 20%/s ramps and 0.1 s for 50%/s ramps.

It would be tempting to interpret the frequency values during concentration ramps simply as being the response to instantaneous odor concentration. For it was already shown that impulse frequency takes on different steady values with different values of steady odor concentration (**Figure 6**, in Burgstaller and Tichy, 2011). However, impulse frequency was also shown not to depend exclusively on odor concentration. Impulse frequency is also governed by the rate with which concentration changes (Figures 3, **5**, in Burgstaller and Tichy, 2012). A comparison of the responses of the ON and OFF ORNs to concentration ramps of different rates confirms this observation. Figure 3 illustrates the responses of a pair of ON and OFF ORNs to a series of three alternating upward and downward concentration ramps in a trapezoid fashion, each ramp covering a concentration range of roughly 100%. A ramp sequence always began with rates of +5%/s and −5%/s. This was followed by ramps of +20%/s and −20%/s, and then completed by ramps of +50%/s and −50%/s. At slow ramps (+5%/s), the ON-ORN's frequency increase peaked slightly after the concentration plateau, but at faster ramps (+20 and +50%/s) the frequency maxima occurred closer to the beginning of the plateau phase. The faster the upward ramp, the faster and the higher frequency increased. During the concentration plateau, the ON-ORN's discharge rate gradually decreased. The slower the upward ramp, the sooner this decrease began and the lower impulse frequency was at the end of the concentration plateau. Like the frequency increase during the upward ramps, the ON-ORN's frequency decrease to falling odor concentration was faster at the faster ramps (−20 and −50%/s) than at the slow ramp (−5%/s). The response decrease of the OFF ORN to upward ramps was faster the faster concentration was falling. During the concentration plateau, the discharge ceased. The cessation period lasted till the end of the plateau phase. At the beginning of the downward ramps, the discharge increase of the OFF ORN was somewhat faster than the discharge decreased of the ON ORN. In addition, the faster the downward ramps, the higher the OFF-ORN's discharge rate.

**Figure 4:**
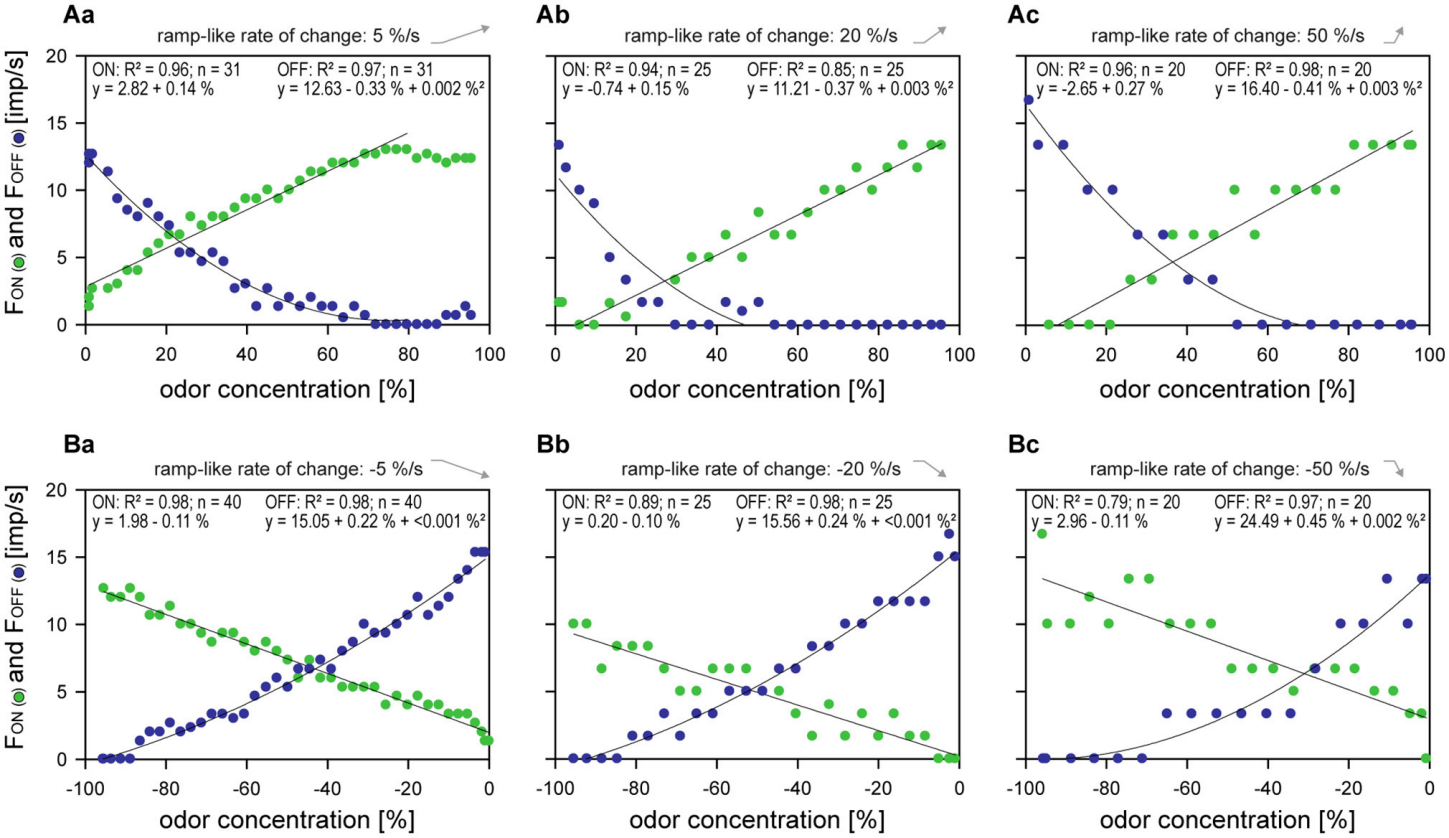
**Responses of an ON ORN (***green***) and an OFF ORN (***blue***) from the same sensillum to a series of three ramp-like upward (Aa–Ac) and downward (Ba–Bc) concentration changes at rates indicated, plotted as functions of instantaneous odor concentration**. Linear regressions were used to approximate the stimulus-response relationships for the ON ORN and parabolic regressions for the OFF ORN. Data points were excluded from analysis if they reach constant values at approaching the end of a ramp; then they represent a new steady state activity yielding zero slope values (ON and OFF ORN in **Aa**; OFF ORN in **Ab** and **Ac**). The negative value for differential sensitivity reflects the downward direction of concentration change yielding a rise in impulse frequency and specifies the OFF ORN. Bin widths for impulse counts were 0.5 s for 5%/s ramps, 0.2 s for 20%/s ramps and 0.1 s for 50%/s ramps. *R*^2^, coefficient of determination.

An estimation of the sensitivity of the ON and OFF ORNs for ramp-like changes in odor concentration will be provided by relating their responses to the instantaneous odor concentrations associated with the concentration ramps. Sensitivity does not refer here to threshold but rather to gain (i.e., change in impulse frequency per unit change instantaneous concentration), and is described by the slope of each curve used to approximate the stimulus-response function. The curves are linear regressions for the ON ORNs and for the OFF ORNs parabolic regressions because they yield a better fit. Figures 4A,B illustrates the functions for a single ON and OFF ORNs. The functions indicate an increase of the response scatter about their characteristic curves with increasing rate of concentration change. The faster the rate of change, the larger was the variability of the responses of the ON and OFF ORN to upward (Figure 4A) and downward ramps (Figure 4B). Such an increase in the response variability might be expected if the magnitude of the responses becomes larger and larger by increasing the rate of change. However, this is not the case. The frequency range covered by an individual ORN is quite similar for the different ramps. It is therefore likely that the increase in response variability is the result of the increasing rate of concentration change.

**Figure 5:**
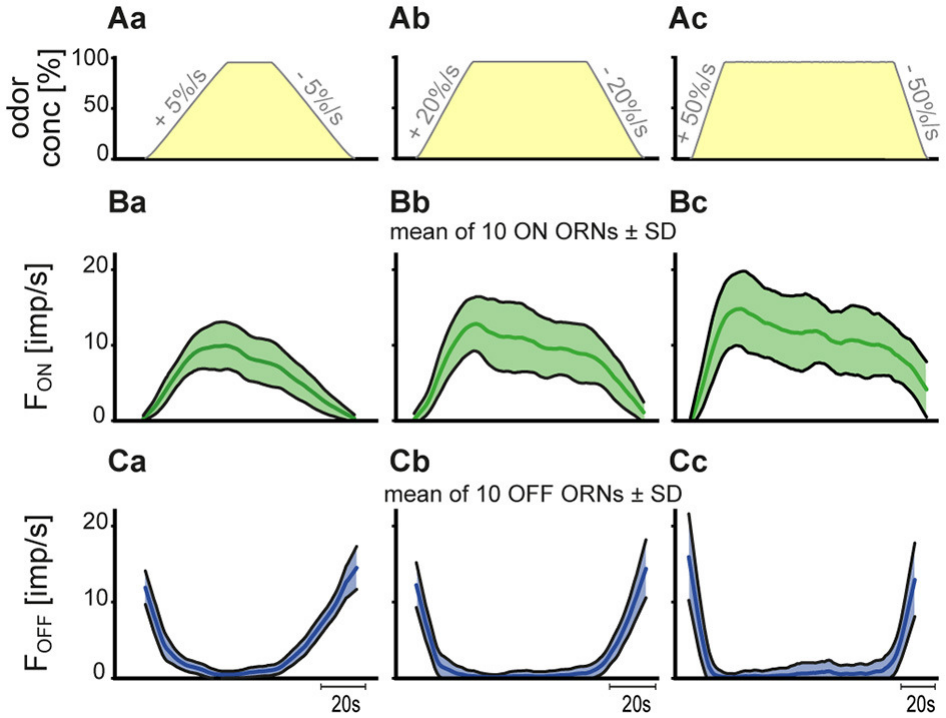
**(A–C)** Time course of pooled responses of 10 pairs of ON and OFF ORNs from the same sensilla to three ramp-like upward and downward concentration changes. **(A)** time course of the three upward and downward concentration ramps. **(B,C)** mean response functions providing a group estimate of the time course of response. Shaded bands illustrate standard deviations of responses from the mean response function. Bin widths for impulse counts were 0.5 s for 5%/s ramps, 0.2 s for 20%/s ramps, and 0.1 s for 50%/s ramps.

The cockroach's capacity to resolve incremental changes of slowly rising or falling odor concentration is not based on the responses of a single ON and OFF ORN. Rather, discrimination depends on the input from a population of the ORNs responding to the particular stimulus. The simplest way of combining the responses of individual ORNs in order to provide a single-response measure is to average them. The curves in Figures 5B,C illustrate the average time course of the responses and the standard deviation of the mean for a population of 10 ON and 10 OFF ORNs during the three series of upward and downward concentration ramps (Figures 5Aa–Ac). The mean population response was taken from the individual responses of the 10 ORNs obtained at the same value of instantaneous odor concentration. The cumulative evidence from the pooled data shows that the impulse frequency of the ON ORN during upward ramps tended to attain higher frequency values at the higher than at the lower ramp rates (Figures 5Ba–Bc). Importantly, both the response magnitude and the response variability increased with rising ramp rates, as indicated by the larger standard deviations of the responses about the mean curves. The pooled data of the OFF ORN, in contrast, display less response variability due to the smaller standard deviations of the responses about the mean curves (Figure 5Ca–Cc). A comparison of the mean response curves for upward and downward ramps indicates that the ON and OFF ORNs are not merely mirror images. The more steeply sloping stimulus-response functions of the OFF ORNs and their smaller standard deviations imply greater information content.

A drawback to the pooling illustrated by Figures 5Ba–Bc is the relatively large deviation of the ON-ORN's responses about the mean function used to approximate their course. Even when the range of the function is narrowed and limited to the upward or downward ramps, and when new functions are calculated for the remaining data points, the standard deviation about the corresponding function is almost the same. More importantly, the scatter for the pooled data is greater than that shown by any single ORN. This is because the scatter results not only from the deviations of the responses from the characteristic curves of individual ORNs. It is equally if not more the product of variance in the slopes of these curves. The effect of the difference of the slope and position of the individual characteristic curves on the pooled responses can be eliminated. This involves first determining the deviation of individual responses from the individual characteristic curves and then treating all the deviations as though they belong to a single ORN of differential sensitivity obtained by averaging the individual slope values. The slopes of the ON ORNs were calculated by linear regressions, those of the OFF ORNs, due to the better fit, by parabolic regressions for individual series. The differential sensitivity (the change in impulse frequency in response to concentration ramp when instantaneous concentration is changed by a constant amount) is less simply specified by parabolic than linear regressions. This is because the slope—and hence the differential sensitivity—varies continuously over the range of instantaneous concentrations. Therefore, the slope value was calculated for each instantaneous concentration used as a stimulus by taking the first derivative of the associated parabola. The combined values from the 10 parabolas of single upward and downward ramps were then averaged. Means and standard deviations of the slope values for each concentration ramp were calculated and used to determine the resolving power (Table 1).

**Table 1:** **Summary of data used to determine differential sensitivity and resolving power of ON and OFF ORNs**.

**1. ON OLFACTORY RECEPTOR NEURON**					
A. Stimulus: rate of upward ramps	+5%/s		+20%/s		+50%/s
a. Number of ORNs tested extensively	20		20		20
b. Number of ORNs used for linear regressions	10		10		10
c. Number of points per linear regression	>35		>20		20
d. Mean slope of linear regressions, *imp/s*	0.16 ± 0.02	^***^	0.17 ± 0.02	^***^	0.20 ± 0.02
e. Mean deviations of responses, *imp/s*	0.08 ± 0.21	^**^	0.09 ± 0.02	^**^	0.11 ± 0.02
f. Coefficient of determination, *R^2^*	0.99 ± <0.01		0.98 ± 0.01		0.97 ± 0.01
g. Resolving power, *%*	8.19 ± 1.55	^***^	11.14 ± 1.99	^***^	14.87 ± 2.68
B. Stimulus: rate of downward ramps	−5%/s		−20%/s		−50%/s
a. Number of ORNs tested extensively	20		20		20
b. Number of ORNs used for linear regressions	10		10		10
c. Number of points per linear regression	>35		>20		20
d. Mean slope of linear regressions, *imp/s*	0.09 ± 0.01	^***^	0.12 ± 0.02	^***^	0.2 ± 0.02
e. Mean deviations of responses, *imp/s*	0.05 ± 0.04	^**^	0.13 ± 0.03	^**^	0.15 ± 0.02
f. Coefficient of determination, *R^2^*	0.98 ± 0.01		0.92 ± 0.03		0.97 ± 0.01
g. Resolving power, *%*	11.55 ± 2.16	^***^	23.26 ± 5.13	^***^	85.23 ± 24.21
**2. OFF OLFACTORY RECEPTOR NEURON**					
A. Stimulus: rate of upward ramps	+5%/s		+20 %/s		+50 %/s
a. Number of ORNs tested extensively	20		20		20
b. Number of ORNs used for parabolic regressions	10		10		10
c. Number of points per parabolic regression	> 35		> 20		20
d. Mean slope of parabolic regressions, *imp/s*	−16.32 ± 3.22	^***^	−21.96 ± 3.95	^***^	−24.11 ± 7.93
e. Mean deviations of responses, *imp/s*	0.02 ± 0.52	^**^	0.08 ± 0.06	^**^	0.15 ± 0.17
f. Coefficient of determination, *R^2^*	0.90 ± 0.03		0.94 ± 0.02		0.96 ± 0.02
g. Resolving power, *%*	5.55 ± 4.00	^***^	7.51 ± 1.41	^***^	9.00 ± 2.09
B. Stimulus: rate of downward ramps	−5%/s		−20 %/s		−50 %/s
a. Number of ORNs tested extensively	20		20		20
b. Number of ORNs used for parabolic regressions	10		10		10
c. Number of points per parabolic regression	>35		>20		20
d. Mean slope of parabolic regressions, *imp/s*	−9.16 ± 3.37	^***^	−12.34 ± 4.92	^***^	−14.94 ± 6.56
e. Mean deviations of responses, *imp/s*	0.08 ± 0.05	^**^	0.21 ± 0.25	^**^	0.27 ± 0.47
f. Coefficient of determination, *R^2^*	0.98 ± 0.01		0.97 ± 0.01		0.95 ± 0.04
g. Resolving power, *%*	5.95 ± 1.34	^***^	8.98 ± 2.39	^***^	13.20 ± 5.89

*a, ORNs employed for studies of the ramp rate as well as the effect of ramp amplitude (not analyzed here). b, ORNs used for finding the best fitting curve to the observed responses. c, bin width for 5%/s ramps was 0.5 s, resulting in 40 points for the 20 s ramp; bin width for 20%/s ramps was 0.2 s, resulting in 25 points for the 5 s ramp; bin width for 50%/s ramps was 0.1 s, resulting in 20 points for the 2 s ramp. Data points were excluded from analysis if they reach constant values at approaching the end of a ramp; then they represent a new steady state activity yielding zero slope values. d, differential sensitivity was determined by the slopes of linear regressions for ON ORNs and parabolic regressions for OFF ORNs. Because in parabolic regressions the slope of the curve varies continuously over the concentration range, the slope value was found for each instantaneous concentration used to determine impulse frequency, by taking the first derivative of the associated parabola. The mean and standard deviation of the slope values were calculated. The negative value for differential sensitivity reflects the downward direction of concentration change yielding a rise in impulse frequency and specifies the OFF ORN. e, mean and standard deviation of individual responses from characteristic curves. f, coefficient of determination indicates the proportion of variance in the dependent variable that is predictable from the independent variable. g, resolving power is the difference in two concentration values discriminable with 90% probability by single ON or OFF ORNs at average differential sensitivity. P probability from t-test that the two neighboring values are different*:

** *P < 0.01*,

*** *P < 0.001*.

The resolving power of an ORN is an estimate of the amount by which two instantaneous concentrations must differ in order for a single ON or OFF ORN of average differential sensitivity to be able to distinguish them with a specified high degree of probability (e.g., 90%). The basis for the distinction: a single response to each of the two concentrations. The demand placed on it: that the higher impulse frequency be associated with the higher concentration in the ON ORN and the lower concentration in the OFF ORN. The resolving power was calculated as described in Material and Methods. The basic data are shown in Table 1. The calculations indicate that the faster the rate of concentration change was, the poorer the resolving power of both ORNs tended to become. The resolving power of the ON ORN for upward ramps was 8% for ramps of +5%/s, 11% for ramps of +20%/s, and only 14% for ramps of +50%/s (Table 1, 1Ag). The corresponding values of the OFF ORN were slightly better: 5, 7, and 9% (Table 1, 2Ag). The resolving power of the OFF ORN for downward ramps was 5% for ramps of −5%/s, 8% for ramps of −20%/s, and only 13% for ramps of −50%/s (Table 1, 2Bg). The corresponding ON ORN values were inferior: 11, 23, and 85% (Table 1, 1Bg).

## Discussion

We describe here for the first time the ability of the cockroach's ON and OFF ORNs to resolve increments and decrements of continuously rising and falling odor concentrations (provided by linear upward and downward concentration ramps). The resolving power depends on the differential sensitivity, which is approximated by the slope of the stimulus-response functions, as well as on the reliability of the response, which is indicated by the scatter of individual points about these functions.

The differential sensitivity of the ON and OFF ORNs for upward and downward concentration ramps is inversely related to response reliability. As the rate of upward concentration ramps rises, the differential sensitivity of both ORNs increases (Table 1, 1Ad, 2Ad). With increasing differential sensitivity, however, the scatter of responses about the stimulus-response functions also increases (Table 1, 1Ae, 2Ae). The greater differential sensitivity is compensated by a growing scatter. Therefore, the resolving power of the ON and OFF ORNs for concentration increments diminishes with rising rate of upward concentration change (Figure 1, 1Ag, 2Ag). Similar results are observed in the reverse situation. As the rate of downward concentration ramps rises, the differential sensitivity of the ON and OFF ORNs increases (Table 1, 1Bd, 2Bd). However, the increase in differential sensitivity is degraded by the decrease in response reliability (Table 1, 1Be, 2Be). The resolving power of both ORNs for concentration decrements therefore diminishes with rising rate of downward concentration change (Figure 1, 1Ag, 2Ag). Since a finite amount of time is available to collect and process sensory information, fast rates of change are beyond the upper limit for accurately determining odor concentration.

**Figure 6:**
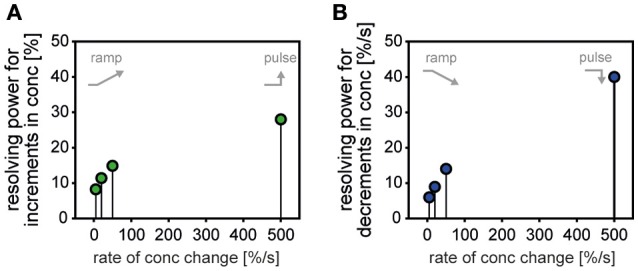
**(A,B)** Comparison of the resolving power of ON ORNs (*green*) and OFF ORNs (*blue*) for slow and continuous, ramp-like upward and downward concentration changes with the resolving power of both types of ORNs for abrupt, step-like upward and downward concentration changes. **(A)** The resolving power of the ON ORN for concentration increments decreases with rising rate of upward concentration ramps and decreases even further when the rate of change rises 10-fold due to abrupt, step-like upward concentration changes. **(B)** The resolving power of the OFF ORN for concentration decrements decreases with rising rate of downward concentration ramps and decreases even further when the rate of change rises 10-fold due to abrupt, step-like downward concentration changes. Data for step-like concentration changes from Burgstaller and Tichy (2011).

The tendency for the precision of the ON and OFF responses to deteriorate with a rising rate of upward and downward concentration ramps is corroborated by the resolving power of both ORNs for step-like upward or downward concentration changes (Burgstaller and Tichy, 2011). Even though the rate of change could not be measured during a step-like concentration change, 100 ms is the estimated transition time. This period was needed to change the mixing ratio between the clean and the odor-saturated air streams. During the first 100 ms of the rapid concentration change, a 50% upward concentration step would result in an average rate of change of 500%/s, and a 100% upward step would produce an average rate of even 1000%/s. In the ON ORN, the resolving power for a +50% upward concentration step, producing an estimated rate of change of +500%/s, was only 28% compared to 8% for the +5%/s ramp and 14% for the +50%/s ramp (Figure 6A). In the OFF ORN, the resolving power for a −50% downward concentration step, with an estimated rate of change of −500%/s, was only 40% compared to 6% for the −5%/s ramp and 14% for the −50%/s ramp (Figure 6B).

The individual ON and OFF ORNs have very limited ability to distinguish between the different rates at which concentration changes. In the ON ORN, the frequency ranges elicited by the three upward ramps differed only minimally. In the OFF ORNs, the ranges for the three downward ramps are also similar to each other. Moreover, both ORNs display a double dependence on the instantaneous odor concentration and the rate of concentration change. This does not imply, however, that they are incapable of providing the CNS with useful information on both parameters. The reason is that the two ORNs are not a symmetrically responding set of receptors; the stimulus-response functions of the ON ORN for upward ramps are never mirror images of the OFF-ORN's functions for downward ramps. Thus, handling the output of both ORNs simultaneously may enable separating the rates of concentration change quantitatively. As the number of receptors increases, errors from response variation should become smaller (Duchamp-Viret and Duchamp, 1997; Duchamp-Viret et al., 1998). Therefore, the CNS may perceive more precise odor concentration values than observed from single responses. In principle, central processing can affect the resolving power in two ways. First, for a fixed peripheral input, any variability contributed by the central neurons may increase the resolving power. Second, any nonlinearity between the peripheral and final neural representation on which discrimination is based may change the sensitivity of this representation for concentration ramps. The variability of the central neural events may increase with the magnitude of the peripheral neural input, such that its standard deviation is a linear function of that peripheral response measure. Thus, when more neural activity is generated, the discriminative task becomes noisier. In the cockroach's antennal lobe, however, may neurons have a lower activity than the ORNs (Hellwig and Tichy, unpubl. results).

The observation that the ON and OFF ORNs provide more accurate information about increments and decrements in odor concentration when the rate of changes is slow suggests that they can detect slow concentration changes when tracking an odor path. This property would allow the cockroach to use information about pulse onset and offset slopes in addition to the pulse height to encode the spatial-temporal structure of turbulent odor plumes (Moore and Atema, 1988, 1991; Zimmer-Faust et al., 1995; Finelli et al., 1999). Intuitively, the inverse relationship between the rate of concentration change and resolving power is not surprising because accuracy requires time. In behavioral studies, the two common measures are speed and accuracy, and the increase in accuracy with the time taken to produce a behavioral response is known as the speed-accuracy tradeoff (Klein, 2001; Uchida and Mainen, 2003; Heitz, 2014). In principle, this speed-accuracy tradeoff is an adaptive process managing the balance between making decisions correctly while not wasting time. This balance has been studied for decades in humans and has been observed in the behavior of many other animals, ranging from rats to bees (Heitz, 2014). Olfactory perception in mammals is characterized by sequential sampling locked to the respiratory cycle. In rats, discrimination of two pure odorants and their mixtures is only minimally affected by odor sampling time: the maximum accuracy was achieved in less than 200 ms, corresponding to just one sniff. More sniffs did not improve the accuracy of odor quality discrimination (Uchida and Mainen, 2003). By contrast, vibro-tactile detection accuracy in rats improves with extended stimulus sampling time (McDonald et al., 2014). Likewise, visual discrimination of images of natural objects by rats is better in slow trials with longer reaction time than in fast trials with shorter reaction time (Reinagel, 2013).

The observation that the ON and OFF ORNs perform more accurately in discriminating concentration changes if the rate of change is slow corresponds with the results from rats in image discrimination (Reinagel, 2013) or vibro-tactile detection tasks (McDonald et al., 2014). Longer sample time, or the longer the stimulus is present within the time window utilized for stimulus detection, lead to more accurate representation. This points to a time-dependent improvement in concentration detection. Cockroaches might finely discriminate changes in odor concentrations as long as concentration changes slowly. At high rates, however, they will detect the presence of the odor signal while foregoing accurate concentration estimation. This speed-accuracy tradeoff represents an economic compromise between confidence and sampling time. It entails an acceptable error with potentially enormous saving in time.

Cockroaches are able to locate odor sources that emit turbulent plumes. Thus, the antennal ORNs no doubt help extract specific spatial information from the rate at which the intermittent odor signal arrives (Willis and Avondet, 2005; Willis et al., 2008; Lockey and Willis, 2015). Due to their poor performance in estimating the concentration of transient odor pulses, the flow direction of the wind bearing the odor plume (rather than the concentration gradient of the pulses) could be used to steer toward the odor source. As the plume ages, the turbulent dispersal changes the values of the plume parameters, i.e., the slopes become less steep and their height falls. By spatially comparing these changes, animals can estimate source and distance information (Zimmer-Faust et al., 1988, 1995; Moore and Atema, 1991). Cockroaches, which have an earth reference such as contact cues of the ground, may extract distance information from gradients in pulse slope and pulse concentration. Distance information could be used in deciding whether to approach the source (if it is energetically worthwhile) or staying put. The greater the distance to the plume source, the higher the probability that competitors may have already found and exploited the source.

In an ecological context, the dynamic responses of ORNs should match those dynamic features of an odor plume that contain important biological information (Moore and Atema, 1988). The variation of synchronization properties between the ON and OFF ORNs suggests that each ORN type has an inherent dynamic response capability. The ability to experimentally record the activity of the ON and OFF ORNs simultaneously allows a clear conclusion: the observed response differences were not due to stimulus variability but were a characteristic property of the ORNs. It has been argued that the differences in the spectral sensitivity and in the stimulus-response function of individual hydroproxoline-sensitive neurons in the lobster aesthetasc chemoreceptors provide a mechanism for encoding stimulus quality as well as stimulus concentration in different across-fiber patterns (Derby and Atema, 1988; Johnson et al., 1991; Gomez et al., 1999). The differences in both the direction and the rate of change of the discharge of the ON and OFF ORNs suggest that the cockroach may likewise encode the upward and downward rates of concentration changes by across-fiber patterns. The capability of the ON and OFF olfactory system to perform such an analysis of odor pulses would depend on the central connections. If information about the rate of change can be extracted by glomeruli in the antennal lobe based on specific innervations, then the olfactory system can rapidly temporally analyze the dynamic features of the odor plume by using across-fiber patterns (Gomez et al., 1994, 1999). Knowing the range of rates of concentration changes detected by the ON and OFF ORNs and their accuracy in resolving increments and decrements in odor concentration may now allow us to recognize the features of odor pulses that contribute to plume tracking.

In conclusion, this study was designed to explore the ORNs ability to detect and resolve slow changes in odor concentration. The values of resolving power are unique to date. Other terrestrial arthropods have still to be examined in this regard. Also unique is the finding that the resolving power depends on the rate with which concentration changes. The greater precision for slow and continuous concentration changes in comparison to rapid and abrupt concentration changes may indicate environmental priorities in odor processing. Because of experimental constraints the range of rates of concentration changes has not been explored adequately in insect olfactory systems. There is an experimental bias for rapid concentration changes, whereby the rate of concentration change was not considered as stimulus parameter. Rapid concentration changes have traditionally been used in electrophysiological experiments to imitate the patchy, intermittent structure of turbulent odor plumes in electrophysiological experiments. Intermittency, however, is a dimensionless value defined as the proportion of the periods in which odor concentration is below threshold (odor absent) to above threshold (odor present) (Cardé and Willis, 2008). With this definition, the possible effect of the magnitude of instantaneous odor concentration and the rate of concentration change were excluded from analysis. In order to find the upper limit of detection of intermittent odor signals, the EAG response of the antennae of the honey bee and locust was tested for its ability to follow brief pulses of 2-heptanone. By approaching a maximum pulse frequency of 125 Hz, odor transduction was proposed to occur within less than 2 ms (Szyszka et al., 2014).

We were interested in the ORNs ability to detect and process creeping changes in odor concentration. A sequence of one linear upward ramp of +5%/s followed by one linear downward ramp of −5%/s will take 40 s. This period corresponds with a frequency of 0.025 Hz which is 5 orders of magnitude lower than the maximum pulse frequency being detected by EAG responses of the honey bee and locust (Szyszka et al., 2014). The ON-ORN's mean differential sensitivity for a +5%/s ramp was 0.16 imp/s (Table 1Ad); therefore, a change in impulse frequency by 1.3 imp/s will be able to differentiate a concentration change of 8.2% which is the ON ORN's resolving power for upward concentration ramps (Table 1Ag). Systematic examination of encoding the rate of concentration change at higher levels of olfactory processing would present an exciting opportunity toward understanding how temporal features of the odor stimulus have shaped the performance of the cockroach's olfactory system.

## Author contributions

LMZ, MH, and HT conceived and designed experiments; LMZ and MH performed experiments and analyzed data; MH and HT interpreted results and wrote the paper; MH prepared figures; HT edited and revised manuscript.

### Conflict of interest statement

The authors declare that the research was conducted in the absence of any commercial or financial relationships that could be construed as a potential conflict of interest.
